# *In vitro* antagonistic potential of *Trichoderma* species against phytopathogenic fungi isolated from selected vegetable crops

**DOI:** 10.3389/fmicb.2026.1845088

**Published:** 2026-09-08

**Authors:** Habibu Bashir Labaran, Zafar Sultan, Dalhatu Wada Taura

**Affiliations:** 1 Department of Biological Sciences, Rabiu Musa Kwankwaso College of Advanced and Remedial Studies, Tudun Wada, Kano State, Nigeria; 2 Department of Biological Sciences, Northwest University, Kano, Kano State, Nigeria; 3 Department of Microbiology, Bayero University, Kano, Kano State, Nigeria

**Keywords:** biological control, dual-culture assay, fungal phytopathogens, mycoparasitic potential, *Trichoderma harzianum*

## Abstract

*Trichoderma* species are capable of causing significant changes in the metabolic activities of host plants, thereby triggering plant growth and increasing plant defense mechanisms against a diverse variety of fungal infections. This species has been reported as potential bio-control agents of many phytopathogenic fungi. Therefore, this study aimed to assess the *in vitro* antagonistic activity of indigenous *Trichoderma* spp. isolated from selected locations across Kano State, Nigeria, against phytopathogens affecting selected vegetables, in a dual-plate assay. Molecular characterization revealed three *Trichoderma* isolates: *T. koningii* (accession number KY886143.1; 99.63% BLAST similarity), *T. harzianum* (KJ807357.1; 98.94%), and *T. viride* (OP546802.1; 98.12%). The results revealed that *Trichoderma* spp. inhibited the radial growth of isolated phytopathogenic fungi compared to the control. The percentage growth inhibition for *T. harzianum* was higher in the tested species. Specifically, the inhibition percentages of *T. harzianum* ranged from 10.82 to 72.32%, for *T. koningii* from 5.49 to 63.15%, and for *T. viride* from 2.22 to 47.33% in the dual-culture assay. The results for *Trichoderma* spp. showed a significant decrease in radial growth and a statistical difference (*p* < 0.05) in the percentage inhibition of the tested pathogens using a one-way ANOVA. The results of this study reveal valuable knowledge on the mycoparasitic potential of *Trichoderma* spp. that could be used in the selection of suitable biological control agents. They also provide useful insights into phytopathogen interactions, relevant to developing strategies for the biological control of fungal phytopathogens in crops of agricultural importance.

## 1 Introduction

Vegetable farming is an essential part of agriculture that provides income, employment, and nutrition while aiding to balance the demand for food security (Dias, 2012). This practice involves the cultivation of edible plant parts such as leaves, stems, roots, flowers, or fruits. Common vegetables include tomatoes, peppers, onions, okra, carrots, spinach, lettuce, cabbage, and cucumbers, among others. Their importance is evident both globally and within local communities, particularly in developing countries where access to nutrition and income opportunities are often limited. In countries such as Nigeria, the local importance of vegetables extends beyond nutritional and economic benefits; they are deeply rooted in culture, health practices, and rural livelihoods (Harriette et al., 2022). Promoting vegetable production and consumption should therefore be a priority for policymakers, researchers, and development partners striving for food security and sustainable development. Despite their importance, vegetable farming faces a wide range of challenges that vary depending on location, scale of production, and method of cultivation. A large fraction of these challenges is attributed to the biotic components of the ecosystem, which primarily include living organisms such as *Fusarium* spp., *Rhizoctonia* spp., *Alternaria* spp., *Aspergillus* spp., and *Meloidogyne* spp., among others—all of which are disease-causing pathogens (Yaseen et al., 2025). These pathogens cause diseases at various stages, from seeds to fully mature plants and in some cases, they continue to affect the crops even after harvest, resulting in substantial losses in both quality and quantity.

Plant diseases pose significant threats to global food security, jeopardizing crop yields and necessitating innovative approaches to disease management and crop protection. Therefore, disease management strategies have evolved rapidly to incorporate innovative strategies for fortifying crops against various pathogens (Kumar et al., 2023). The innovative strategies offer insights into the critical significance of diseases and pioneering approaches for their effective management (Asif et al., 2023). The host–pathogen relationship between plants and fungi is a fundamental biological interaction; how this relationship is allowed to progress in different situations depends on agricultural practices. More than 19,000 species of fungi that cause diseases in crops are documented worldwide (Jain et al., 2019). Tyskiewicz et al. (2022) reported that a majority of the phytopathogens belong to the phyla Ascomycota and Basidiomycota, with some of the most serious pathogens being representative members of the genera *Cladosporium*, *Botrytis*, *Alternaria*, *Aspergillus*, *Verticillium*, *Pythium*, *Fusarium,* and *Rhizoctonia*.

Traditional disease management strategies rely solely on practices such as the application of chemical pesticides, cultural practices, and crop rotation to control plant diseases. While these methods have been effective, they often come with major drawbacks, including environmental pollution, pesticide resistance, and the disruption of natural ecosystems (Tilman et al., 2002). The damage caused by the continuous use of chemical pesticides has shifted research priorities toward innovative strategies that offer natural alternatives, such as biocontrol agents, to steer agricultural production systems toward innovative sustainability. To tackle environmental and public health concerns, various innovative strategies have been adopted for sustainable food production systems, including integrated pest management (IPM), organic farming, and the use of biological control agents (BCAs), among others (Grasswitz, 2019).

Biological control refers to the use of living organisms (known as antagonists or biological control agents, BCAs) to control pests such as insects, weeds, parasites, pathogenic fungi, and other invasive species. While there are numerous examples of extremely effective and environmentally benign BCAs, the broad biodiversity of pest species necessitates continuous innovation in biological control strategies (Stiling and Cornelissen 2005; Yolanda et al., 2023). In addition, BCAs may have undesired side effects on non-target organisms within an ecosystem, underscoring the need for researchers to thoroughly investigate and understand the interactions between potential BCAs and microbial populations in different soils and crops before their widespread commercial use (Massart et al., 2015; Qin et al., 2019). Despite its many complexities, the concept of biological control has become increasingly important in recent years due to public health concerns regarding the need to phase out or reduce the use of traditional chemical-based pesticides in agricultural practices (Wang et al., 2015). This shift has prompted the development of new classes of fungal-based BCAs, such as *Trichoderma* spp., *Aspergillus niger*, and *Ampelomyces quisqualis*, and bacterial-based BCAs, such as *Bacillus* spp., *Agrobacterium radiobacter*, and *Pseudomonas fluorescens*, all of which have demonstrated high efficacy against multiple plant pathogens, with the potential to reduce environmental contamination and delay the development of rapid resistance within target pathogens (Keswani et al., 2016). One of the most widely researched BCAs is *Trichoderma*, which includes ubiquitous mesophilic fungi characterized by an outstanding ability to colonize different environments, and as a result, these fungi can be found in nearly all soil habitats (Yolanda et al., 2023). Therefore, this study aimed to determine the *in vitro* antagonistic potential of *Trichoderma* species against isolated fungal phytopathogens of selected vegetable plants in gardens located in Kano State, Nigeria.

## 2 Materials and methods

The present study was carried out at the Biological Sciences laboratory, Department of Biological Sciences, Northwest University Kano, located at the coordinates 12^o^ 0′ 17’ N, 8^o^ 28′ 47′E Kano State, Nigeria.

### 2.1 Soil sample collection

Rhizospheric soil samples were collected from various commercially grown vegetable fields and gardens in Dambatta, Tudun Wada, Gaya, Gwarzo Local Government Area (LGA), and the botanical garden of Northwest University, Kano State. Ten samples were randomly collected from each location, and soil analysis was conducted at the Laboratory of Biological Sciences Department from August to October 2025. At each sampling location, clean and sterile polyethylene bags were used for collecting representative soil samples. The samples were obtained from a depth of 0 to 10 cm, as described by Chiroma et al. (2014).

#### 2.1.1 Isolation and characterization of *Trichoderma* species from soil samples

Fungal species were isolated from soil samples using the serial dilution plating method, as described by Aliyu et al. (2022). One gram (1 g) of each soil sample was suspended in 10 mL of distilled water (initial working suspension). The solution was then diluted in a series of five vials (10^−1^ to 10^−5^) containing 9 mL of sterile distilled water. A measure of 1 mL of the working suspension was transferred to the first vial using a pipette. Subsequently, 1 mL of the solution from the first vial was transferred to the second vial, and the process was repeated for each subsequent vial. Dilutions (10^−3^ to 10^−5^) were prepared and plated in triplicate onto Petri dishes containing molten potato dextrose agar (PDA) and were incubated at 25 ± 2 °C for 3–5 days. The plates were observed for distinct fungal colonies that appeared after the incubation period in the mixed cultures. Individual colonies were picked and sub-cultured onto fresh PDA plates to obtain pure cultures of each fungus. Pure culture colony types were obtained and maintained on separate plates for further experimentation. Colonial characteristics based on the morphology of the various isolates were noted, and the sticky tape method (Flegel, 1980) was adopted for microscopic identification using a calibrated phase-contrast microscope. The shape and size of the spores and mycelial width of each isolate were recorded, and the pictorial atlas for the identification of fungi by Watanabe (2002) was used. Each species of *Trichoderma* was identified separately as described by Manjur and Afiya (2019) and Aliyu et al. (2022).

### 2.2 Molecular characterization of fungal isolates

Isolates showing features suggestive of *Trichoderma* were further subjected to molecular identification. The molecular analysis of the isolates was conducted at the Department of Microbiology, Bayero University, Kano. The molecular approach ensures accurate species identification.

#### 2.2.1 DNA extraction

Genomic DNA extraction was conducted using a hybrid method, which comprises lysis with a commercially available DNA extraction kit (Qiagen) and the traditional phenol–chloroform method for purification, as described by White et al. (1990). Initially, the isolates were sub-cultured on potato dextrose broth (PDB) and incubated at 37 °C for 18 h. After incubation, the content was centrifuged at 10,000 rpm for 5 min, the supernatant was discarded, and 200 μL of an extraction buffer from the kit (GDSBio, China) and 20 μL of proteinase K were added to the cell suspension. This mixture was left to incubate at 65 °C overnight. Subsequently, the phenol–chloroform extraction process was initiated by adding 200 μL of a phenol: chloroform: isoamyl alcohol (25:24:1) solution to the mixture. The mixture was centrifuged at 12,000 rpm for 10 min, thereby separating the aqueous and organic phases. The upper aqueous phase, which contained DNA, was meticulously collected using a pipette and transferred to a new 1.5-μL microcentrifuge tube. The DNA was then precipitated by adding 100 μL of cold 70% ethanol, followed by centrifugation at 12,000 rpm for 10 min, yielding a DNA pellet. This pellet was washed with ethanol to eliminate impurities. After air-drying to remove any remaining ethanol, the DNA pellet was dissolved in distilled water. Finally, the DNA was stored at −20 °C for preservation.

### 2.3 PCR amplification

The amplification of the internal transcribed spacer (ITS) region of the 18S rRNA genes was carried out on the isolates. The PCR mixture was prepared by adding 2 μL of each extracted DNA, 10 μL of ZymoTaq PreMix (2x) (Master Mix), 1 μL each of the universal primers ITS1 (ITS1 Forward) (5′-TCCGTAGGTGAACCT GCGG-3′) and ITS4 (Reverse) (5′-TCCTCCGCTTATTGATATG C-3′), which target the 18S rRNA gene, and 6 μL of nuclease-free water in a 0.2-μL PCR sterile tubes on ice. The PCR reactions were set up individually for each sample using the following parameters:

- Initial denaturation at 95 °C for 5 min,
- Denaturation at 95 °C for 30 s
- Annealing at 58 °C for 30 s
- Extension at 72 °C for 30 s
- Repetition of steps 2–4 for 30–35 cycles; and
- Final extension at 72 °C for 5 min.

### 2.4 Gel electrophoresis

The success of PCR amplification and the presence of specific amplicons were verified through agarose gel electrophoresis. The procedure began by preparing a 1.5% agarose gel. This involved dissolving agarose powder in 1 × Tris-acetate-EDTA (TAE) buffer, followed by heating the mixture in a microwave oven. To aid in visualizing the gel under UV light, ethidium bromide (2 μL) was added to the molten agarose solution. The gel was poured into a gel-casting tray and allowed to solidify, with comb slots placed at one end to form wells for sample loading. As the gel had solidified, the comb was gently removed, leaving wells ready for loading the PCR products. Then, a sample of 4 μL of the PCR products were loaded into these wells. Electrophoresis was then conducted at 100 V until the DNA bands migrated sufficiently for analysis and were visualized using an ultraviolet (UV) transilluminator.

#### 2.4.1 DNA sequencing and analysis

PCR products from 18S rRNA sequencing with the expected size were purified and sent for Sanger sequencing using the specific universal primers used for amplification at Inqaba Biotech Ibadan, Oyo State, Nigeria. The obtained sequencing data were analyzed using bioinformatics tools. Sequences were compared against public databases (NCBI GenBank) to identify the closest matching species. The results were validated based on sequence similarity and taxonomic classification.

#### 2.4.2 Isolation and identification of fungal pathogens from infested vegetable plants

Vegetable seedling parts (leaves, stems, and roots) of tomatoes, peppers, and okra that exhibited symptoms of disease, such as wilting, leaf spots, or damping-off, were collected for the study. The plant samples were collected in properly labeled, sterilized paper bags, stored, and transported to the laboratory for further analysis, as described by Magaji et al. (2024).

Following collection, the plant samples underwent processing for the isolation and identification of pathogenic fungi responsible for the observed disease symptoms, as described by Magaji et al. (2024). The plant tissues, including roots, stems, and leaves, were surface-sterilized to eliminate external contaminants by dipping the samples in 70% ethanol for 1 min, then soaking them in 2% sodium hypochlorite solution, and rinsing with sterile distilled water. This procedure ensures that only the fungi associated with the internal plant tissues are isolated. The sterilized plant tissues were cut into small pieces and placed onto potato dextrose agar (PDA) in Petri dishes. The Petri dishes were incubated at 25 ± 2 °C for 3–7 days until fungal colonies became visible. Distinct fungal colonies that appeared after the incubation period were picked and sub-cultured onto new plates to obtain pure cultures. Fungal isolates were identified morphologically using colony appearance, microscopic characteristics under a light microscope, and the pictorial atlas for identification of fungi by Watanabe (2002). Lactophenol cotton blue was used to prepare a microscopic slide smear of each isolate for visualization of fungal features and proper identification under the microscope. These structural features were matched with those reported by Akinyemi and Liamngee (2018) and Pahnwar et al. (2023).

#### 2.4.3 *In vitro* antagonistic activity on dual-culture assay

Following the results obtained from stage 1, only the fungal strains identified as *Trichoderma* species were selected to proceed with the experiment. A dual-culture assay was used as described by Nofal et al. (2021). The *in vitro* antagonistic activities of *Trichoderma* isolates were assessed against all the isolated fungal pathogens. Petri dishes (9 cm) containing PDA medium were inoculated with mycelial discs (0.5 cm in diameter) from each isolated pathogen. Then, 5-cm mycelial plugs of either *Trichoderma* isolates were placed opposite the pathogen, while the isolated fungal pathogens were inoculated alone and served as the control treatment. For each treatment, triplicate samples were maintained and incubated for 5–7 days at 27 ± 2 °C in an incubator. Mycelial growth of each fungal pathogen was measured using a ruler and recorded. The percentage inhibition was calculated with respect to the control. The percentage of radial growth inhibition (% RGI) was calculated using the equation:


$$\frac{\left(\mathrm{\% RGI = C}-T\right)}{C}\;X\;100$$

Where C is the radial growth of the tested fungal pathogen without the antagonist and T is the radial growth of the tested fungal pathogen in dual culture with the antagonist.

The ability of *Trichoderma* spp. to overgrow and inhibit the growth of the pathogens was scored according to the scale of Aamir et al. (2019) and modified based on the percentage inhibition as follows:

**Table:** 

S/N	Scale	Interpretation	Description
1	0%	Inhibition not effective	**−**
2	>0.5–19%	Inhibition slightly effective	**+**
3	20–39%	Inhibition moderately effective	**++**
4	40–59%	Inhibition effective	**+++**
5	60–100%	Inhibition very effective	**++++**

#### 2.4.4 Data analysis

The data were subjected to a one-way analysis of variance (ANOVA), and treatment means were compared using Tukey’s test in GENSTAT software version 17.1.

## 3 Results and discussion

### 3.1 Isolation and characterization of antagonist fungi (*Trichoderma* species)

**Figure 1:**
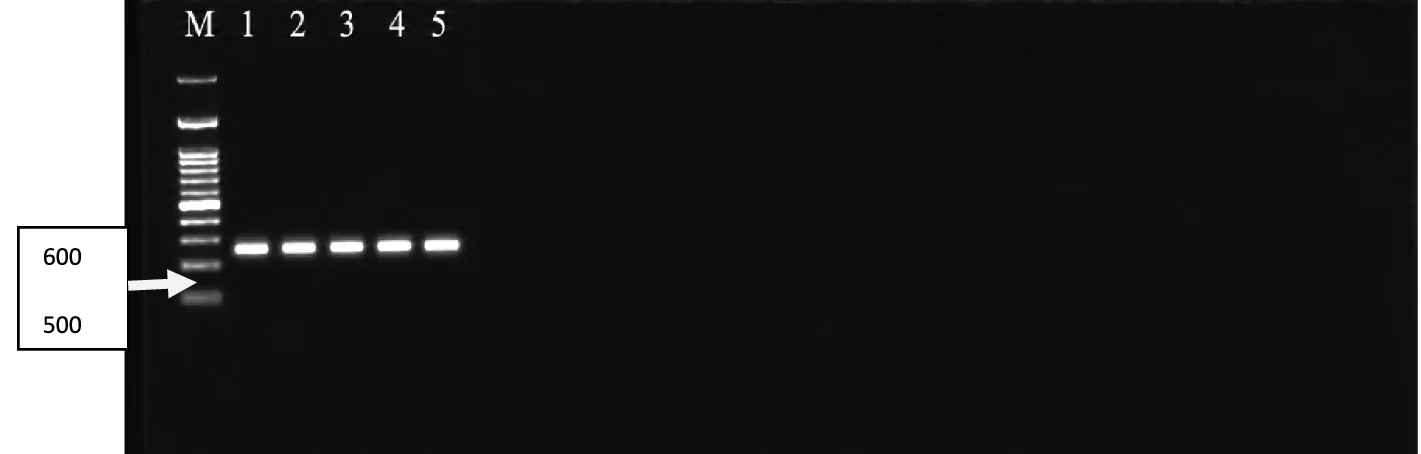
PCR amplification of the ITS gene in five *Trichoderma* isolates.

**Figure 2:**
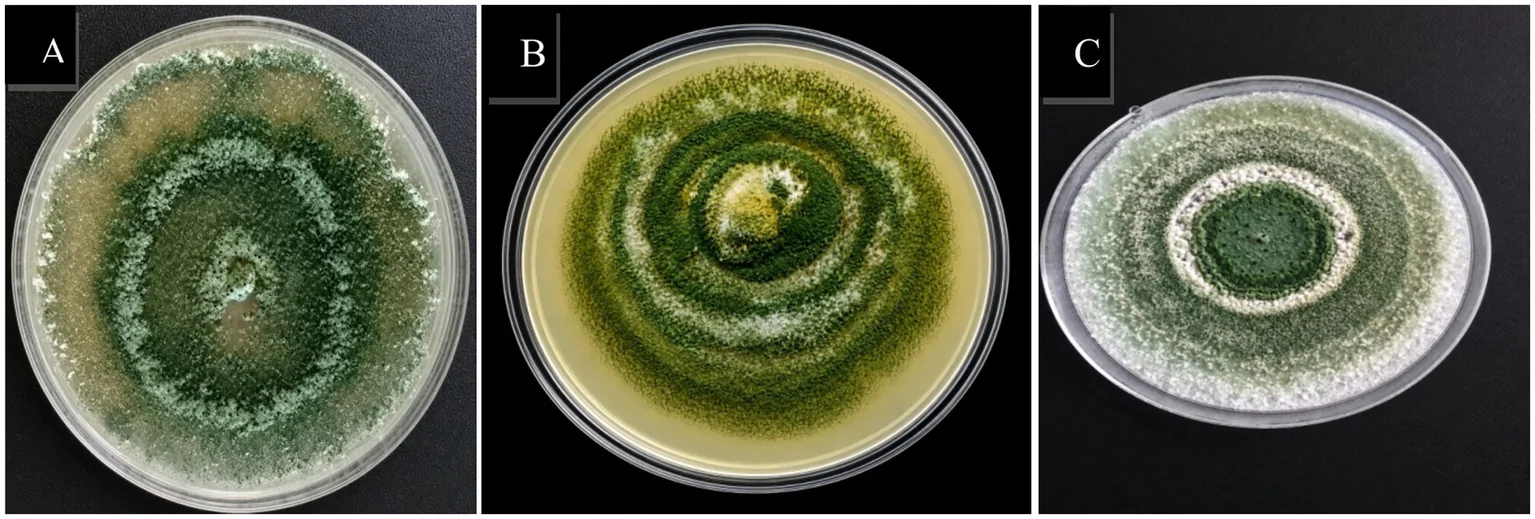
Cultural characteristics of identified *Trichoderma* spp. isolated on PDA. **(A)** isolate 1A, **(B)** isolate 2A, and **(C)** isolate 3A.

Table 1 shows the morphological characteristics and molecular characterization of the 18S rRNA gene (Figure 1) identified from three *Trichoderma* species isolated in soil samples. The results show the *Trichoderma* species used in the research and their cultural features on PDA, namely: *T. koningii*, *T. harzianum,* and *T. viride* (Figure 2). Morphological features were consistent with those reported by Singh et al. (2015), Aliyu et al. (2022), Nwaichi and Nduka (2023), and Kose et al. (2024).

**Table 1:** Morphological and BLAST analysis of *Trichoderma* sp. isolated from soil.

Sample I.D.	Identifiable phenotypes on PDA microscopic characteristics	Species	BLAST search similarity	Accession Number
1A	Dark green colony with a whitish ring-like zone. Irregular branched conidiophores, with the shape of globose conidia.	*Trichoderma viride*	98.12%	OP546802.1
2A	Fast-growing colony of approximately 5–8 cm in 5 days. Light green conidia distributed throughout, forming concentric rings. Distinct septate, regularly branched conidiophore, with the shape of subglobose conidia.	*Trichoderma harzianum*	98.94%	KJ807357.1
3A	Green surface, separate, distinct septate, regularly branched conidiophore, with the shape of subglobose conidia.	*Trichoderma koningii*	99.63%	KY886134.1

### 3.2 Isolation and identification of fungal pathogens from infested vegetable plants

The results, with reference to the isolation and identification of fungal pathogens associated with collected vegetables, viz., pepper, okra, and tomato, revealed the occurrence of different fungal species. A total of seven fungal species, including *Alternaria alternata*, *Alternaria solani*, *Aspergillus flavus*, *Aspergillus niger*, *Fusarium oxysporum*, *Penicillium* sp., and *Rhizopus stolonifer*, were isolated and identified from the tomato plant. Similarly, seven fungi, viz., *A. alternata*, *Aspergillus tenuissima*, *A. flavus*, *A. niger*, *C. capsici*, *Penicillium* sp., and *R. stolonifer*, were isolated and identified from pepper plants. In contrast, six fungi, viz., *A. alternata*, *A. flavus*, *A. niger*, *F. solani*, *Penicillium* sp., and *R. stolonifer*, were isolated and identified from okra plants (Table 2).

**Table 2:** Different fungal species isolated and identified from tomato, pepper, and okra plant parts.

S/N	Fungi species		
	Tomatoes	Pepper	Okra
1	*Alternaria alternata*	*Alternaria alternata*	*Alternaria alternata*
2	*Alternaria solani*	*Aspergillus flavus*	*Aspergillus flavus*
3	*Aspergillus flavus*	*Aspergillus niger*	*Aspergillus niger*
4	*Aspergillus niger*	*Penicillium* sp.	*Penicillium* sp.
5	*Fusarium oxysporum*	*Rhizopus stolonifer*	*Rhizopus stolonifer*
6	*Penicillium* sp.	*Colletotrichum capsici*	*Fusarium solani*
7	*Rhizopus stolonifer*	*Alternaria tenuissima*	-

**Figure 3:**
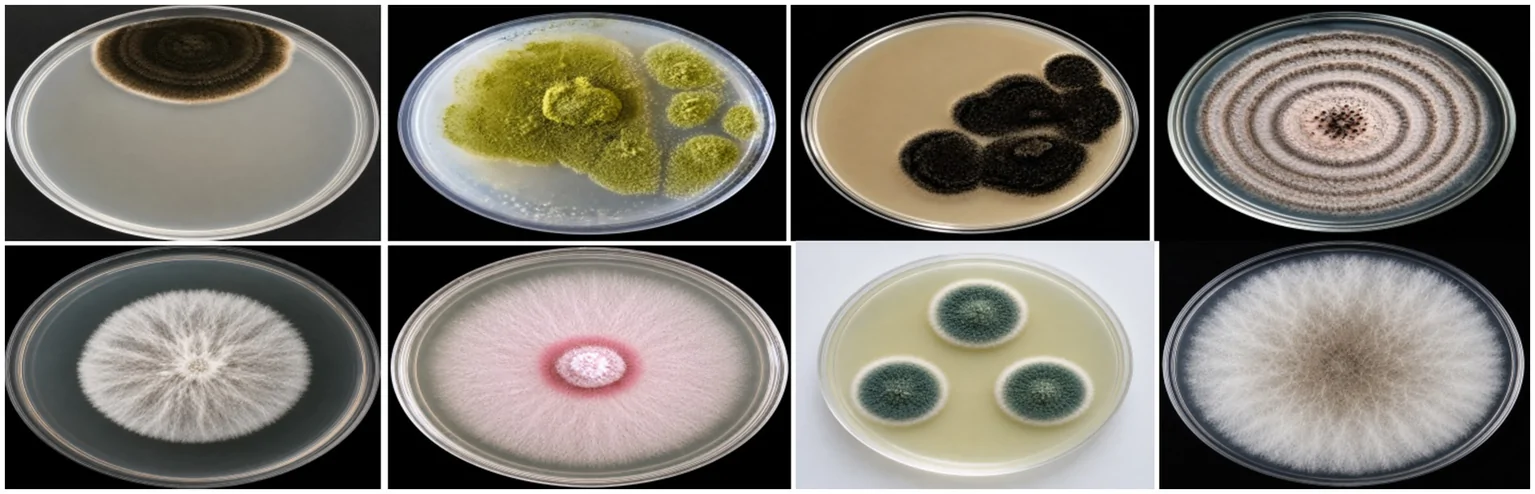
Plates showing colony characteristics on PDA of fungal pathogens isolated from selected vegetable samples.

The results revealed that different diversities of fungi were represented among the fungal pathogens of vegetable plants and exhibited a variety of symptoms in different plant structures. The symptoms were observed on the leaves, internal stem structure (vascular system), roots, and fruits of the collected infected plants. Several fungi are classified as phytopathogenic agents that display a wide range of infectious symptoms, resulting in damage to the plants and affecting their general productivity from germination to the post-harvest stage. Studies have reported damages and economic losses resulting from infestations of these fungal pathogens, including the studies of Hlaiem et al. (2023), Villegas et al. (2024), and Magaji et al. (2024). Such fungal pathogenic agents were identified in our study and belonged to different families, such as Ascomycetes (8), Basidiomycetes (1), and Mucoromycota (1). The identification of plant pathogens was based on the characteristic colony morphology of each fungus on PDA. The features were compared with identification keys reported by Akinyemi and Liamngee (2018) and Pahnwar et al. (2023). The features are presented in Table 3 and Figure 3.

**Table 3:** Morphological characteristics of fungi isolated from infected tomato, pepper, and okra plant parts.

Sample I.D.	Identifiable features on PDA and microscopic characteristics	Suspected species
1A	Cottony mycelia and circular growth ranging in color from whitish to pink shades. Conidia were present. Aseptate mycelium	*Fusarium* spp.
1B	Slow-growing mycelia in shades of blue-green on PDA, irregular, and slightly raised toward the center. Conidiophores branched into brush-like structures.	*Penicillium* sp.
1C	Fast-growing circular colonies on PDA, yellow-brown color in color, with rough-walled conidia	*Aspergillus* spp.
2A	Circular, woolly, or cottony colonies with characteristic pale brown color. Slightly curved conidia were present. Chlamydospores were absent.	*Colletotrichum* spp.
2B	Colony was flat, circular, woolly, and covered with greyish aerial hyphae.	*Alternaria* spp.
2C	White to grey or dark color, cottony, fluffy colonies, and rapid growth, with root-like structures (rhizoids)	*Rhizopus* spp.

### 3.3 *In vitro* antagonist effect of identified *Trichoderma* spp. against the isolated fungal pathogens

*In vitro* assay using *T. koningii*, *T. harzianum,* and *T. viride* against the isolated phytopathogenic fungi showed different responses. Table 4 shows the overall reduction in radial growth of the tested pathogens relative to that in the control plates for each *Trichoderma* species. Mycoparasitic effects were more pronounced with *T. harzianum*, followed by *T. koningii*, with *T. viride s*howing the least effect. The isolated *Alternaria* species (T1, T2, and T9) responded in a similar pattern. However, *Aspergillus niger* (T4) was much reduced compared to *Aspergillus flavus* (T3). *Fusarium* species (T5 and T10) responded similarly and exhibited the greatest radial growth reduction rate among the other pathogens. The isolated *Penicillium* species (T6), *Rhizopus stolonifer* (T7), and *Colletotrichum capsici* (T8) all showed reduced radial growth in the treatment plates than in their control plates. Significant statistical differences (*p* ≤ 0.05) were observed in the radial growth of all the fungal pathogens tested in this study following treatment with the identified *Trichoderma* spp.

**Table 4:** Effects of *Trichoderma* spp. on radial growth (cm) of isolated fungal pathogens of selected vegetables at 7 days after inoculation.

Treatments/pathogens	Control	*T. viride*	*T. koningii*	*T. harzianum*	LSD
T1	5.93^a^	5.00^b^	4.80^b^	4.20^c^	0.26
T2	5.93^a^	4.93^b^	4.83^b^	4.23^c^	0.25
T3	8.30^a^	5.70^b^	5.60^b^	5.23^c^	0.14
T4	8.33^a^	5.67^bc^	5.80^b^	5.20^b^	0.19
T5	6.97^a^	4.20^b^	3.17^c^	2.33^d^	0.31
T6	5.37^a^	4.50^b^	4.60^b^	4.03^c^	0.09
T7	7.53^a^	5.67^b^	5.47^b^	5.20^b^	0.21
T8	5.20^a^	3.83^b^	3.63^b^	3.23^c^	0.15
T9	5.93^a^	4.83^b^	4.77^b^	4.23^c^	0.27
T10	6.97^a^	4.33^b^	4.10^b^	2.33^c^	0.35

*Means followed by the same letter(s) in each row are not significantly different at a *p*-value of ≤0.05. Key: T1 = *Alternaria alternata;* T2 = *Alternaria solani*; T3 = *Aspergillus flavus*; T4 = *Aspergillus niger;* T5 = *Fusarium oxysporum;* T6 = *Penicillium* sp. T7 = *Rhizopus stolonifer;* T8 = *Colletotrichum capsici;* T9 = *Alternaria tenuissima;* T10 = *Fusarium solani.*

**Figure 4:**
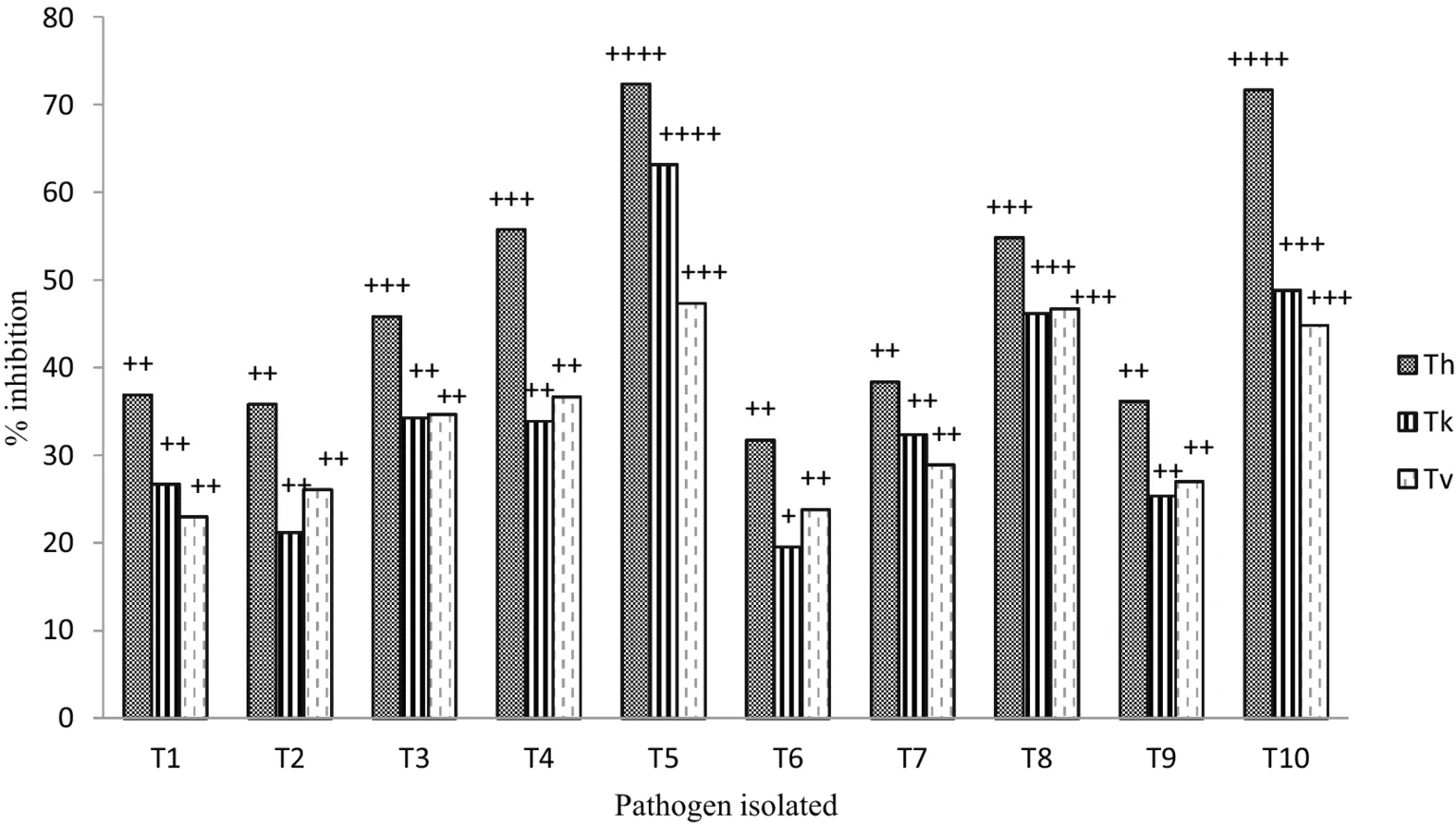
Percentage inhibition of *Trichoderma harzianum* (Th)*, Trichoderma koningii* (Tk), and *Trichoderma viride* (Tv) on mycelial growth of the isolated fungal pathogens after incubation. Key: T1 = *Alternaria alternata*; T2 = *Alternaria solani*; T3 = *Aspergillus flavus*; T4 = *Aspergillus niger*; T5 = *Fusarium oxysporum*; T6 = *Penicillium* sp.; T7 = *Rhizopus stolonifer*; T8 = *Colletotrichum capsici*; T9 = *Alternaria tenuissima*; T10 = *Fusarium solani*.

Figure 4 shows the percentage (%) inhibition of *Trichoderma* spp. against the isolated fungal pathogens. The results revealed that the highest percentage of inhibition was recorded for *T. harzianum,* followed by *T. koningii* and *T. viride*, which showed the least inhibition. The ability of each *Trichoderma* species to overgrow and inhibit the growth of the pathogens was calculated and interpreted according to the scale of Aamir et al. (2019). Moreover, the results revealed that *T. harzianum* exhibited pronounced activity against pathogens T5 and T10 (72.32 and 71.68%, respectively), which interprets as very effective (++++), followed by T4, T8, and T3 (55.76, 54.83, and 45.81%, respectively), which was effective (+++) while other pathogens (T1, T2, T6, T7, and T9) fell within the moderately effective inhibition scale (++), with T6 showing the least response at 31.72%. Significant differences (*p* ≤ 0.05) were obtained for the percentage inhibition of mycelial growth of the fungal pathogens isolated in this study using *T. harzianum*.

The percentage inhibition of *T. koningii* ranged from 5.49 to 63.15%, which was lower than those observed in *T*. *harzianum*. Inhibition percentages were highest with T5, T10, and T8 (63.15, 48.82, and 46.18%, respectively), which were also within the inhibition effective range (+++). T1, T2, T3, T4, T7, and T9 fell within the moderately effective inhibition scale (++), with T6 showing the least effect at 19.51%. Significant differences (*p* ≤ 0.05) were also shown in the percentage inhibition of mycelial growth of the fungal pathogens isolated in this study using *T. koningii* (Figure 4).

Finally, the percentage inhibition by *T. viride* ranged from 2.22 to 47.33%, which was lower than those observed in both *T. harzianum* and *T. koningii*. Inhibition percentages were highest with T5, T8, and T10 (47.33, 46.68, and 44.81%, respectively), which were also within the inhibition effective (+++) range. Other percentages fell within the moderately effective (++) range, i.e., 36.65, 34.66, 28.89, 26.97, 26.05, 23.78, and 22.96, representing T4, T3, T7, T9, T2, T6, and T1, respectively. However, our studies showed that the highest inhibition percentages were observed for *Fusarium* species and *Colletotrichum capsici*, with all *Trichoderma* spp. used.

*Trichoderma* is a genus of the family Hypocreaceae known for its ability to inhibit phytopathogenic fungi. *Trichoderma koningiopsis* has been declared as a biocontrol agent against several pathogens, such as *Sclerotinia asari*, *Fusarium* species, *Verticillium dahliae*, and oomycete pathogens (Kong et al., 2022; Liu et al., 2022; Olowe et al., 2022; Wang et al., 2022). *Trichoderma lixii*, the teleomorph of *T. harzianum*, has been reported as a biocontrol agent that produces antifungal molecules that inhibit the growth of fungi (Elgorban et al., 2014). Research conducted by Chauiyakh et al. (2024) on three pathogenic fungi (lignivorous fungi) exhibited different resistance behavior toward *Trichoderma koningiopsis*, *T. lixii*, *Trichoderma simmonsii,* and *Trichoderma viridescens,* with a maximum colonization percentage not exceeding 75–81.25%. Usman et al. (2023), in their study of the suppressive abilities of *Trichoderma* sp. against *Rhizopus stolonifer,* reported varying antagonistic potential, with percentage inhibition ranging from 66.7 to 87.5% using *Trichoderma reseei*, *T. harzianum,* and *T. koningii* in sweet potatoes. Similarly, Aliyu et al. (2022) concluded that three species of *Trichoderma* (*Trichoderma harzianum*, *Trichoderma viride,* and *Trichoderma asperellum*) inhibited the mycelial growth of *Fusarium oxysporum f.* sp. *vasinfectum* in dual culture to varying degrees, with *Trichoderma harzianum* being the most effective, followed by *Trichoderma viride* and finally *Trichoderma asperellum*. *Trichoderma* species can act as biocontrol agents against fungal phytopathogens via a variety of mechanisms, such as competition for nutrients and space, antibiosis, induction of plant defense mechanisms, and mycoparasitism (Ferreira and Musumeci, 2021). When selecting biological control agents, the confrontation plate assay is frequently used as a preliminary test. It enables the study of hyperparasitism, the effects of non-volatile metabolites, and nutrient competition. The *Trichoderma* species tested showed varying degrees of antagonism via different mechanisms involved in inhibiting the mycelial growth of the isolated fungal phytopathogens.

Researchers have reported the antagonistic activity of *Trichoderma* species against a variety of pathogen agents (Aamir et al., 2019; Aliyu et al., 2022; Hlaiem et al., 2023). It is worth noting that the tested *Trichoderma* spp. showed a strong antagonistic relationship, although higher responses were recorded with *Trichoderma harzianum* than the others. Inhibition of pathogens shown by antagonists may be due to the release of antibiotics or substances similar to secondary metabolites or the parasitism of hyphae, which results in direct inhibition of the growth of the pathogen by integrating the hyphal wall, resulting in penetration, absorption, and lysis of mycelium (Asif et al., 2023). Another study attributed the inhibition of radial growth to the secretion of extracellular lytic enzymes, the production of antibiotic compounds, and cell wall-degrading enzymes such as chitinase and glucanase, which degrade polysaccharides, chitins, and glucans, thereby destroying cell wall integrity (Kong et al., 2022). These enzymes may play a key role in mycoparasitism by altering cell wall integrity prior to physical contact. *Trichoderma* isolates can invade and sporulate on colonies of pathogens. The inhibitory effect may also be due to chemicals released by *Trichoderma* isolates during antibiosis. Indeed, several studies have reported the importance of molecules released by *Trichoderma* spp. in their antagonistic action against pathogenic fungi (Kong et al., 2022). *In vitro* tests confirmed the efficacy of volatile metabolites produced by different *Trichoderma* species in controlling the growth of phytopathogenic fungi.

## 4 Conclusion

In this research, three *Trichoderma* species were successfully isolated and identified from soil samples associated with vegetable gardens: *Trichoderma viride*, *T. harzianum*, and *T. koningii*. Their morphological characteristics were consistent with established identification criteria, while molecular/BLAST analysis showed high sequence similarity of 98.12–99.63% with reference sequences. However, a diverse range of fungi was associated with the infected vegetable plants. A total of 10 fungal species were identified across tomato, pepper, and okra, including *Alternaria alternata*, *A. solani*, *A. tenuissima*, *Aspergillus flavus*, *A. niger*, *Fusarium oxysporum*, *F. solani*, *Penicillium* sp., *Rhizopus stolonifer*, and *Colletotrichum capsici*. The fungal pathogens were associated with different plant parts and disease symptoms, indicating that fungal infections can affect several stages and structures of vegetable plants, including leaves, stems, roots, and fruits, with potential consequences for crop growth and productivity. All three *Trichoderma* species demonstrated antagonistic activity against the tested fungal pathogens *in vitro*. Treatments with the *Trichoderma* isolates consistently reduced the radial growth of the pathogens compared with the untreated controls. *Trichoderma harzianum* exhibited the strongest overall antagonistic activity. It produced the greatest reduction in radial growth for most of the tested pathogens and recorded the highest percentage inhibition, demonstrating superior biocontrol potential among the three *Trichoderma* species evaluated. *Trichoderma koningii* showed intermediate antagonistic activity, generally performing better than *T. viride* but less effectively than *T. harzianum*. *Trichoderma viride* showed the lowest overall antagonistic activity among the three species. Nevertheless, it still demonstrated appreciable inhibitory effects against several of the tested pathogens, particularly *Fusarium* spp. and *C. capsici*. The differences among treatments were statistically significant (*p* ≤ 0.05) for radial growth and percentage inhibition, indicating that the observed inhibitory effects were not merely due to random variation. The effectiveness of *Trichoderma* was pathogen-dependent, with the degree of inhibition varied considerably among fungal species. This finding demonstrates that the antagonistic ability of a *Trichoderma* isolate depends not only on the *Trichoderma* species but also on the particular pathogen being challenged. Overall, the study demonstrates the biocontrol potential of the isolated *Trichoderma* species, with *T. harzianum* emerging as the most promising candidate for further investigation as a biological control agent against fungal pathogens of tomato, pepper, and okra.

## Data Availability

The raw data supporting the conclusions of this article will be made available by the authors, without undue reservation.
